# Altered developmental trajectories of verbal learning skills in 22q11.2DS: associations with hippocampal development and psychosis

**DOI:** 10.1017/S0033291722001842

**Published:** 2023-08

**Authors:** Caren Latrèche, Johanna Maeder, Valentina Mancini, Karin Bortolin, Maude Schneider, Stephan Eliez

**Affiliations:** 1 Developmental Imaging and Psychopathology Lab, Department of Psychiatry, University of Geneva School of Medicine, Geneva, Switzerland; 2 Medical Image Processing Lab, Institute of Bioengineering, EPFL, Lausanne, Switzerland; 3 Clinical Psychology Unit for Intellectual and Developmental Disabilities, Faculty of Psychology and Educational Sciences, University of Geneva, Geneva, Switzerland; 4 Department of Neurosciences, KU Leuven, Center for Contextual Psychiatry, Leuven, Belgium; 5 Department of Genetic Medicine and Development, University of Geneva School of Medicine, Geneva, Switzerland

**Keywords:** 22q11.2 deletion syndrome, developmental trajectories, hippocampus, psychosis, verbal memory

## Abstract

**Background:**

The cognitive profile in 22q11.2 deletion syndrome (22q11.2DS) is often characterized by a discrepancy between nonverbal *vs.* verbal reasoning skills, in favor of the latter skills. This dissociation has also been observed in memory, with verbal learning skills described as a relative strength. Yet the development of these skills is still to be investigated. We thus aimed to explore verbal learning longitudinally. Furthermore, we explored verbal learning and its respective associations with hippocampal alterations and psychosis, which remain largely unknown despite their high prevalence in 22q11.2DS.

**Methods:**

In total, 332 individuals (173 with 22q11.2DS) aged 5–30 years completed a verbal-paired associates task. Mixed-models regression analyses were conducted to explore developmental trajectories with threefold objectives. First, verbal learning and retention trajectories were compared between 22q11.2DS *vs.* HC. Second, we examined hippocampal volume development in 22q11.2DS participants with lower *vs.* higher verbal learning performance. Third, we explored verbal learning trajectories in 22q11.2DS participants with *vs.* without positive psychotic symptoms and with *vs.* without a psychotic spectrum disorder (PSD).

**Results:**

Our findings first reveal lower verbal learning performance in 22q11.2DS, with a developmental plateau emerging from adolescence. Second, participants with lower verbal learning scores displayed a reduced left hippocampal tail volume. Third, participants with PSD showed a deterioration of verbal learning performance, independently of verbal reasoning skills.

**Conclusion:**

Our study challenges the current view of preserved verbal learning skills in 22q11.2DS and highlights associations with specific hippocampal alterations. We further identify verbal learning as a novel cognitive marker for psychosis in 22q11.2DS.

## Introduction

Chromosome 22q11.2 deletion syndrome (22q11.2DS) is a neurogenetic condition associated with an elevated risk of developing psychotic disorders, rendering it a promising genetic model for studying the development of schizophrenia (Weisman et al., 2017). The syndrome is also associated with a specific neuropsychological profile encompassing deficits in many domains such as executive functions and attention (Maeder et al., 2016; Morrison et al., 2020; Schneider et al., 2014b). The cognitive phenotype in 22q11.2DS is generally characterized by impaired nonverbal skills whereas verbal reasoning skills are reported as relatively less affected (Jacobson et al., 2010; Lewandowski, Shashi, Berry, & Kwapil, 2007). Verbal reasoning skills refer to crystallized knowledge, which is involved in understanding and reasoning with verbal concepts (Wechsler, 2014). These skills are typically measured by the verbal intellectual quotient (VIQ). In 22q11.2DS, a discrepancy in VIQ-performance IQ (in favor of the VIQ) has often been reported, especially in school-age children (Margolis et al., 2018; Swillen, 2016; Swillen et al., 1999). Indeed, in a sample of children with 22q11.2DS, Jacobson et al. (2010) found performance IQ scores in the range of mild intellectual disability (*m* = 64.2, s.d. = 8.7), while VIQ scores fell into borderline intellectual functioning (*m* = 72.4, s.d. = 12.8). Interestingly, the dissociation between verbal and nonverbal domains has also been observed in memory (Bearden et al., 2001; Maeder, Zuber, Schneider, Kliegel, & Eliez, 2022; Woodin et al., 2001).

Memory is a dynamic system that includes three core processes: encoding, storage, and retrieval (Squire & Zola-Morgan, 1991), with only encoding and retrieval being directly assessable by standard cognitive tasks. Differences between nonverbal *vs.* verbal abilities were identified in both processes in 22q11.2DS. Bostelmann, Glaser, Zaharia, Eliez, and Schneider (2017) found that impaired visual exploration had a detrimental impact on learning and therefore on retention of visual information. By contrast, verbal memory appears to be relatively preserved (Campbell et al., 2010; Lajiness-O'Neill et al., 2005; Sobin et al., 2005). Indeed, verbal learning is especially recognized as a relative strength in the cognitive profile in 22q11.2DS (Debbané, Glaser, & Eliez, 2008; Lepach & Petermann, 2011). Being less affected, the verbal domain has only been explored by a few studies that carry several limitations. First, relatively small sample sizes were predominantly employed (e.g. Lepach & Petermann, 2011; Sobin et al., 2005), likely due to the prevalence of 22q11.2DS (Blagojevic et al., 2021; Botto et al., 2003; Olsen et al., 2018). Second, the presence of a control group was not systematic across previous research, as participants' performance was compared to psychometric test norms (e.g. Swillen *et al*. 1999; Tobia, Brigstocke, Hulme, & Snowling, 2018). Third, earlier studies mainly examined verbal abilities in specific age groups (children and adolescents *vs.* adults; e.g. Debbané et al., 2008; Fiksinski et al., 2019) instead of treating age as a continuous variable, thereby leaving the development of verbal memory skills largely unknown. This issue raises a fourth limitation, namely that previous work mostly used cross-sectional designs, which do not account for the intra-individual variability described in the cognitive profile in 22q11.2DS (Philip & Bassett, 2011). As 22q11.2DS affects the neurodevelopment, adopting a longitudinal design would allow a better understanding of how verbal learning and subsequently, verbal retention abilities mature over time. Altogether, these findings highlight the need for a more thorough investigation of the developmental trajectory of verbal memory skills in 22q11.2DS.

From a neuroimaging perspective, verbal learning and verbal retention skills rely on the left medial temporal lobe (Kelley et al., 1998) and particularly on the hippocampus (Antoniades et al., 2018). Alterations in this region affect both encoding and retrieval processes (Squire, Stark, & Clark, 2004). For instance, in a hippocampal-dependent task (i.e. concrete verbal-paired associates), people with bilateral hippocampal damage showed poorer performance (Clark, Kim, & Maguire, 2018). In 22q11.2DS, hippocampal abnormalities are well-documented, with a decrease in gray matter volume being commonly reported (Debbané, Schaer, Farhoumand, Glaser, & Eliez, 2006; DeBoer, Wu, Lee, & Simon, 2007; Flahault, Schaer, Ottet, Debbané, & Eliez, 2012; Mancini et al., 2019). In a longitudinal study, Mancini et al. (2019) showed altered patterns of hippocampal development in 22q11.2DS individuals compared to healthy controls (HC). Using a cross-sectional approach, Maeder et al. (2020) compared hippocampal volume between 22q11.2DS participants with lower or higher verbal memory scores. When normalizing retention scores for verbal learning performance, they observed a significant reduction of bilateral hippocampal volume in participants with short- and long-term accelerated verbal memory decline. Yet, to our knowledge, the developmental trajectory of hippocampal volume in relation to memory abilities in 22q11.2DS remains unknown. The use of a longitudinal design would again provide a more accurate understanding of the relationship between hippocampal development and verbal learning.

From a psychiatric perspective, impairments in the verbal domain are well-established in schizophrenia (Owens et al., 2011; Valli, Tognin, Fusar-Poli, & Mechelli, 2012) and typically occur in the context of a broader cognitive decline (Mollon & Reichenberg, 2018). This neurocognitive deterioration is commonly observed several years prior to the onset of psychotic symptoms, with verbal memory deficits being identified before the transition to psychosis in high-risk individuals (Valli et al., 2012). Interestingly, the cognitive profile in idiopathic schizophrenia is often similar to the one described in 22q11.2DS individuals with psychosis. Two cross-sectional studies (Chow, Watson, Young, & Bassett, 2006; Fiksinski et al., 2019) reported severely impaired verbal learning skills (*z* = −3.06 and *z* = −2.76, respectively) in 22q11.2DS adults with *vs.* without psychosis. Nevertheless, identifying developmental trajectories of verbal skills from childhood could provide insight into early predictive factors for psychosis in 22q11.2DS. Accordingly, Vorstman et al. (2015) examined verbal reasoning skills longitudinally, and revealed a steeper decline for the VIQ (9.02 points) in deletion carriers with *vs.* without psychosis. Being apparent from early adolescence, this cognitive decline is recognized as a strong indicator of the development of psychosis. Yet, despite the widely documented deficits in verbal learning abilities in idiopathic schizophrenia (Antoniades et al., 2018), the development of such abilities in relation to psychosis remains unexplored in 22q11.2DS. Therefore, investigating verbal learning skills longitudinally in individuals with *vs.* without psychotic symptoms may represent a novel and more specific marker of psychosis in 22q11.2DS.

The present study, divided into two parts, aims to better understand the development of verbal memory skills in 22q11.2DS in relation to brain maturation and psychosis. In the first study, we aimed to explore the developmental trajectories of verbal learning and verbal retention in 22q11.2DS participants and HC using a longitudinal design. In line with previous studies showing relatively preserved verbal memory skills, we expected to observe similar trajectories between both groups. An additional aim was to study the development of the hippocampus according to verbal learning performance in 22q11.2DS participants. We hypothesized that the developmental trajectory of hippocampal volume would differ between participants with lower *vs.* higher verbal learning performance.

In the second study, we aimed to examine the development of verbal learning in 22q11.2DS participants with *vs.* without psychotic symptoms. We focused on positive psychotic symptoms (PPS) rather than negative symptoms, given their stronger relationship with memory processes (Maeder et al., 2020). Due to the high prevalence of PPS in 22q11.2DS (Schneider et al., 2014a), we also created subgroups of participants with or without a psychotic spectrum disorder (PSD), to determine its role as a possible early cognitive marker of psychosis. Consistently with findings in idiopathic psychosis (Antoniades et al., 2018), we predicted lower verbal learning performance in participants with PPS, and less improvement with age compared to those without PPS. We expected to find more pronounced differences in verbal learning trajectories between participants with *vs.* without PSD.

## Study 1: verbal memory trajectories and associations with hippocampal development

### Method

#### Participants

Participants were recruited in the context of an ongoing longitudinal study based in Geneva (e.g. Maeder et al., 2016). In total, 332 individuals participated in the current study, with 173 having a 22q11.2 deletion confirmed by quantitative fluorescent polymerase chain reaction (QF-PCR). The remaining 159 participants were HC (including 71% siblings and community controls), who underwent screening for psychiatric illnesses and psychotropic medication prior to inclusion. Written informed consent based on protocols approved by the Swiss Ethical Committee of Geneva was obtained from parents or participants.

The participants' age ranged from 5 to 30 years. At inclusion, both groups were matched for age and sex but not for full-scale IQ (FSIQ; online Supplementary material [SM] Appendix 1, Table A1). In total, 740 time-points were acquired, with a significantly greater number for 22q11.2DS (57%) *vs.* HC (*p* = 0.005; Table 1). Time-points ranged from 1 to 6 and were spaced 3.64 years (s.d. = 0.88) apart on average, with a mean of 2.03 time-points per participant.

**Table 1.** Time-points available for 22q11.2DS and HC and psychiatric diagnosis and psychotropic medication per time-point for 22q11.2DS

			Time-points						
			T1	T2	T3	T4	T5	T6	Total
	*N*	22q11.2DS	173	113	69	40	21	3	420
		HC	159	87	47	18	10	0	320
		All	332	200	116	58	31	3	740
Psychiatric diagnosis (%)	Total		127 (73.41%)	74 (64.91%)	44 (38.6%)	28 (70%)	14 (66.67%)	2 (66.67%)	289 (68.81%)
	Categories	Simple phobia	71 (41.04%)	38 (33.63%)	20 (28.99%)	13 (32.5%)	6 (28.57%)	2 (66.67%)	150 (35.71%)
		Attention deficit disorder	66 (38.15%)	31 (27.43%)	15 (21.74%)	3 (7.5%)	4 (19.05%)	0	119 (28.33%)
		Generalized anxiety disorder	30 (17.34%)	16 (14.16%)	12 (17.39%)	8 (20%)	6 (28.57%)	1 (33.3%)	73 (17.38%)
		Major depressive episode	14 (8.09%)	13 (11.5%)	14 (20.3%)	7 (17.5%)	5 (23.81%)	1 (33.3%)	54 (12.86%)
		Social phobia	18 (10.4%)	8 (7.08%)	3 (4.35%)	1 (2.5%)	1 (4.76%)	1 (33.3%)	32 (7.62%)
		Obsessive-compulsive disorder	12 (6.94%)	5 (4.42%)	2 (2.9%)	1 (2.5%)	1 (4.76%)	0	21 (5%)
		Psychosis spectrum disorder	7 (4.05%)	5 (4.42%)	4 (5.80%)	3 (7.5%)	2 (9.52%)	0	21 (5%)
	Total		28 (16.18%)	43 (38.05%)	30 (43.47%)	14 (35%)	12 (54.55%)	2 (66.67%)	129 (30.71%)
Psychotropic medication (%)	Categories	Psychostimulants	11 (6.36%)	25 (21.93%)	16 (14.04%)	7 (6.14%)	2 (9.09%)	1 (33.33%)	62 (14.76%)
		Antipsychotics	15 (8.67%)	14 (12.28%)	9 (7.89%)	4 (3.51%)	8 (36.36%)	2 (66.67%)	52 (12.38%)
		Antidepressants	3 (1.73%)	11 (9.65%)	15 (13.16%)	10 (8.77%)	10 (45.45%)	2 (66.67%)	51 (12.14%)
		Antiepileptic	4 (2.31%)	1 (0.88%)	0	3 (2.63%)	2 (9.09%)	0	10 (2.38%)
		Anxiolytics	1 (0.58%)	3 (2.63%)	2 (1.75%)	2 (1.75%)	1 (4.55%)	0	9 (2.14%)

#### Materials

*Clinical assessment*. All 22q11.2DS participants and their parents underwent a clinical assessment with a trained psychiatrist (SE). For participants under 18 years, their parents were interviewed using the computerized Diagnostic Interview for Children and Adolescents-Revised (DICA-R; Reich, 2000) to detect the presence of DSM-IV psychiatric disorders. Participants from 18 years and their parents were interviewed separately using the Structured Clinical Interview for DSM-IV Axis I (SCID-I; First, Spitzer, Gibbon, & Williams, 1996). Psychotic disorders were assessed with the supplement of the Schedule for Affective Disorders and Schizophrenia for School-Age Children Present and Lifetime Version (K-SADS-PL; Kaufman et al., 1997), completed separately with participants and parents. Psychiatric disorders and psychotropic medication taken at each time-point are reported in Table 1.

*Assessment of verbal learning and verbal retention skills.* Participants were assessed at each time-point with the Verbal Paired Associates (VPA) subtest from a memory scale adapted for their age. Children and adolescents until 16 years and 11 months completed this task in the Children's Memory Scale (CMS; Cohen, 1997) whereas individuals from 17 years and older completed the third or fourth version of the Wechsler Memory Scale (WMS; Wechsler, 1997a, 1997b, 2009). The VPA task was used to assess the encoding (verbal learning) and retrieval (verbal retention) of concrete word pairs. This subtest includes three different subscores for the learning phase, the immediate recall, and the delayed recall. As the number of word pairs and of trials vary depending on the memory scale, learning and retention percentages were computed from raw scores to allow comparability between scale versions (see SM Appendix 2 for details).

*Intellectual functioning.* Intellectual functioning was assessed at each time-point using the Wechsler intelligence scale for children from 6 to 16 years and 11 months and for adults from 17 years (Wechsler, 1991, 1997b, 2008, 2003, 2014). As participants completed different versions of the Wechsler scales given the longitudinal design of the study, only the FSIQ was included.

*Neuroimaging.* T1-weighted brain scans were available in 155 of the 173 22q11.2DS participants (90% of the sample). Due to the longitudinal design of this study, magnetic resonance imaging (MRI) scans were acquired using three different scanners: a 1.5 T Philips Intera scanner, a 3 T Siemens Trio, and a 3 T Siemens Prisma. The parameters for the acquisition of structural images for the T1-weighted MPRAGE sequence were TR = 2500 ms, TE = 3 ms, flip angle = 8°, acquisition matrix = 256 × 256, field of view = 23.5 cm, voxel size = 0.9 × 0.9 × 1.1 mm and 192 slices. T1-weighted images underwent fully automated image processing with the software FreeSurfer version 6.0 (https://surfer.nmr.mgh.harvard.edu), comprising skull stripping, intensity normalization, reconstruction of internal and external cortical surfaces and parcellation of subcortical brain regions (Destrieux, Fischl, Dale, & Halgren, 2010). We analyzed the volume of the whole hippocampus and of the following seven hippocampal subfields: tail, subiculum, granulate cells of the molecular layer of the dentate gyrus (GC-ML-DG), molecular layer, CA1, CA2/3, and CA4 (Iglesias et al., 2015). Quality control procedures of the segmentation were performed as in Mancini et al. (2019, 2021).

#### Statistical analyses

*Descriptive analyses.* Descriptive statistics were performed using Graphpad Prism v.8.0.1 for Macintosh (GraphPad Software, San Diego, CA, USA). Participants' first time-point was used to compare age, gender, and FSIQ between both 22q11.2DS and HC groups.

*Mixed-model regression analyses.* We conducted mixed-model regression analyses using MATLAB R2019b (Mathworks, Natick, MA, USA) as in our previous studies (Franchini et al., 2018; Maeder et al., 2016; Mancini et al., 2019; Mutlu et al., 2013). We first examined the developmental trajectories of verbal learning and verbal immediate and delayed retention in 22q11.2DS *vs.* HC. We then explored developmental trajectories of whole hippocampus and hippocampal subfields volume in 22q11.2DS with higher and lower verbal learning scores. Mixed-modeling is well-adapted for repeated measures, characterized by variable time intervals and inconsistent time-points between individuals (Shaw et al., 2006), as in the case in our longitudinal cohort (e.g. Mancini et al., 2019). The within-subject factor is modeled as a nested variable (Dedrick et al., 2009) while age and diagnosis are modeled as fixed effects. The *nlmefit* function in MATLAB is used to fit several models (constant, linear, quadratic, cubic) to the data and the Bayesian information criterion (BIC) method is then used to select the most suitable model. A likelihood ratio test is performed to assess statistically significant between-group differences in developmental trajectories. Two types of results can emerge from using this analysis: intercept differences (i.e. trajectories showing parallel slopes but different intercept values) and/or slope differences (i.e. trajectories showing different slopes). For verbal learning, a complementary analysis was conducted by covarying for verbal reasoning (measured by standard scores from the Similarities subtest of the Wechsler Intelligence Scales). For hippocampal volume, total gray volume, sex, and scan type were entered as covariates. We reported FDR corrected *p* values and measures of effect size as ß-values for the intercept and the slope in each group (Franchini et al., 2018; Mancini et al., 2021).

To further examine hippocampal volume development in 22q11.2DS, participants were clustered into two subgroups according to their trajectories of verbal learning using MATLAB (SM Appendix 3).

### Results

#### Developmental trajectories of verbal learning and verbal retention

**Fig. 1.**
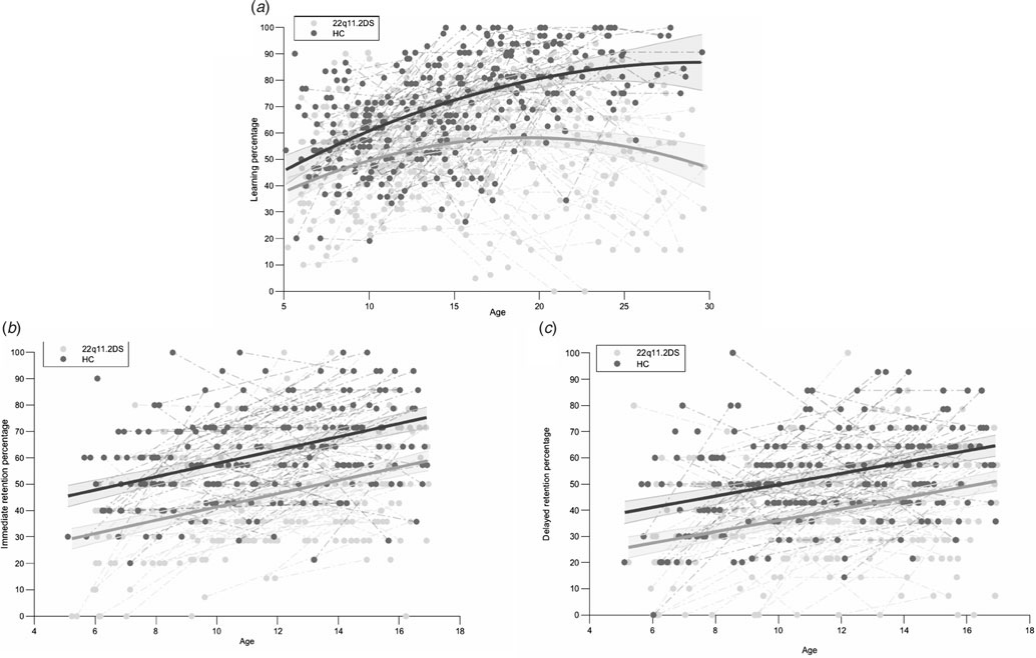
Comparison in developmental trajectories between 22q11.2DS and HC participants for (*a*) verbal learning performance, (*b*) immediate retention performance, and (*c*) delayed retention performance.

Developmental trajectories of verbal learning and of verbal immediate and delayed retention between 22q11.2DS participants and HC are displayed in Fig. 1*a*–*c*, respectively. Verbal learning followed a quadratic model while verbal immediate and delayed retention followed a linear model (SM Appendix 4, Table A3). The three measures exhibited significant intercept differences in the groups' trajectories (*p* < 0.001), indicating lower performance in 22q11.2DS. When the amount of information acquired in the verbal learning phase was considered, differences remained significant for immediate and delayed recalls. Only verbal learning displayed a significant interaction with age (*p* < 0.001), with 22q11.2DS participants improving less with age and reaching a developmental plateau faster than HC. When using verbal reasoning as a covariate for verbal learning, the differences in shape and in intercept remained significant (*p* < 0.001; SM Appendix 5, Fig. A2).

#### Developmental trajectories of hippocampal volume

**Fig. 2.**
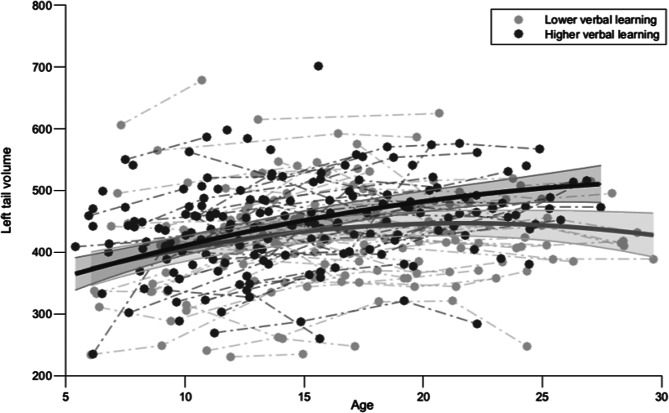
Comparison in developmental trajectories of left tail volume in 22q11.2DS between participants with lower verbal learning performance and participants with higher verbal learning performance.

Developmental trajectories of volume from the whole hippocampus and hippocampal subfields were compared between 22q11.2DS participants with lower and higher verbal learning performance. Statistically significant results were found for the left tail only, showing a quadratic relationship with age (SM Appendix 6, Table A4). The trajectories of this subfield showed significant group differences, revealing lower left tail volume in participants with lower verbal learning performance (*p* < 0.001), and significant interaction with age showed a smaller increase of left tail volume in this subgroup (*p* < 0.001; Fig. 2).

## Study 2: verbal learning development in relation to PPS and PSD in 22q11.2DS

### Method

#### Participants

Only 22q11.2DS participants were included in the second study. First, to explore the presence of PPS in our sample, only participants assessed by the Structured Interview for Psychosis-Risk Syndromes (SIPS) in at least one time-point were considered. Given that the SIPS is not administered to younger children, 41 participants were excluded. In line with Schneider et al. (2014b), we excluded 13 participants below 12 years to reduce the risk of false negatives (i.e. individuals without PPS who might develop symptoms later). The final sample was composed of 119 participants, with 351 assessments in total. To create subgroups, we selected an intensity threshold based on the SIPS scores (Delavari et al., 2021; Mancini et al., 2019). Participants experiencing moderate-to-severe PPS (i.e. score of ⩾3 on any subscale for PPS in at least one time-point) were categorized as PPS + (*n* = 74). The remaining participants were considered PPS-. Both subgroups were commensurate for age, sex, and FSIQ (SM Appendix 7, Table A5). Second, we divided participants according to the presence of PSD into two subgroups: participants with a diagnosis (PSD +; *n* = 19), *vs.* without (PSD-; *n* = 100). Both subgroups were matched for age and sex but differed in FSIQ (SM Appendix 8, Table A6).

#### Materials

*Clinical assessment.* To create PPS + and PPS- subgroups, we used the SIPS, an adapted and validated instrument in 22q11.2DS (Miller et al., 2003; Tang et al., 2014). It consists of symptoms that belong to four subscales (positive, negative, disorganization, and general prodromal). This clinical tool was used to assess the presence and the severity of PPS at each time-point. The positive subscale includes the five following symptoms: Unusual Thought Content (P1), Suspiciousness (P2), Grandiose Ideas (P3), Hallucinations (P4), and Disorganized Communication (P5), each rated on a 7-severity scale by the interviewer (SE). To create PSD + and PSD- subgroups, we used the DICA-R and the SCID-I, previously described in Study 1.

*Assessment of verbal learning skills.* As psychosis is currently understood as the result of abnormal brain development occurring years before the illness onset (Rapoport, Giedd, & Gogtay, 2012), we aimed to focus on a broad age range. Therefore, to explore the trajectories of verbal memory skills in relation to psychosis, we used the learning subscore from the VPA task already presented in Study 1. Unlike the verbal retention assessment, the verbal learning assessment was present and comparable in both the CMS and the WMS, allowing us to explore trajectories from childhood to adulthood (see flowchart in SM Appendix 2, Fig. A1). Results were also covaried for verbal reasoning performance.

#### Statistical analyses

First, descriptive analyses were conducted using Graphpad Prism 80.0 to allow age, gender, and FSIQ comparisons between PPS + and PPS-, and between PSD + and PSD- subgroups. Second, mixed-model regression analyses were performed to compare trajectories of verbal learning between the PPS and the PSD subgroups.

### Results

#### Developmental trajectories of verbal learning in relation to PPS and PSD

**Fig. 3.**
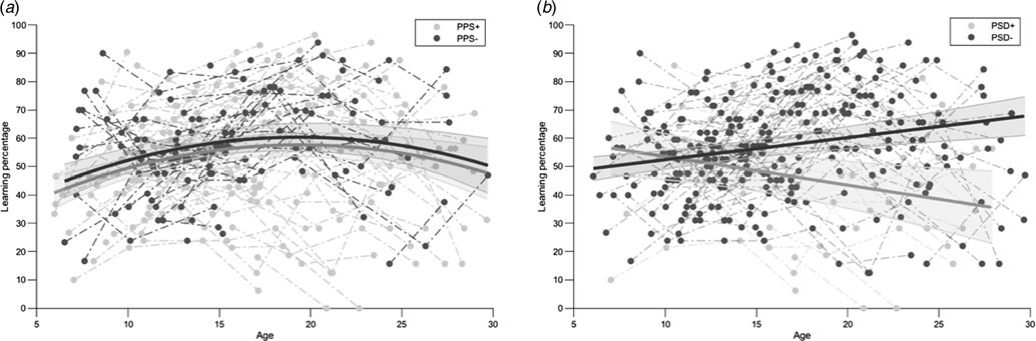
Comparison in developmental trajectories for verbal learning performance between (*a*) PPS + and PPS- subgroups and (*b*) PSD + and PSD- subgroups.

For verbal learning, developmental trajectories between PPS subgroups followed a quadratic model (Fig. 3*a*). Verbal learning showed no significant differences neither in slope (*p* = 0.113) nor in intercept (*p* = 0.150), indicating comparable performance between subgroups (SM Appendix 9, Table A7). Developmental trajectories of verbal learning between PSD subgroups followed a linear model (Fig. 3*b*). Statistically significant differences were detected for the intercept (*p* < 0.001), yielding poorer performance in PSD + participants (SM Appendix 10, Table A8). Regarding interaction with age, verbal learning showed a significant slope difference (*p* < 0.001), revealing divergent trajectories between PSD subgroups. This divergence started between childhood and puberty, at around 11 years old. When covarying for verbal reasoning performance, both significant differences remained for PSD subgroups (*p* < 0.001).

#### Longitudinal correlations of verbal learning performance and PPS severity

We also explored the longitudinal correlations between verbal learning performance and PPS severity. Our findings show that verbal learning performance is significantly and negatively correlated with the P1 subscale (i.e. unusual thought content and delusional ideation) as well as with the sum of the five PPS. The method and the detailed results of this supplementary analysis are available in SM Appendix 11.

## Discussion

The present study aimed at better understanding the developmental trajectories of verbal learning skills in 22q11.2DS and their relationship with hippocampal volume and psychotic symptoms. We administered a word-pair (VPA) task to a large sample of 22q11.2DS participants and HC aged 5–30 years and we analyzed the respective developmental trajectories using a longitudinal design.

### Altered developmental trajectories in 22q11.2DS

We found a significantly poorer verbal learning performance as well as less improvement with age in 22q11.2DS, pointing to developmental lags (i.e. individuals showing growth in absolute ability but lagging behind HC; Chawner et al., 2017). Therefore, these results do not support our hypothesis and contrast with the current view of spared verbal learning skills. Such divergences with our findings are likely due to methodological considerations as, to our knowledge, this study is the first that adopted a longitudinal design in a broad age range sample. Furthermore, we showed lower verbal (immediate and delayed) retention scores in children and adolescents with 22q11.2DS, even after controlling for their learning performance. Again, this finding does not align with results from a recent study demonstrating similar delayed recall performance between 22q11.2DS and HC after correcting for the initial learning rate (Maeder et al., 2021). This discrepancy may originate from differences in material used to assess verbal memory. While we employed a concrete word-pair task, previous studies largely used simpler, single-word memory tasks including the CVLT-C (Delis, Kramer, Kaplan, & Ober, 1994) and adaptations from the RAVLT (Rey, 1958). Stimulus complexity indeed appears to be of relevance when studying verbal memory in 22q11.2DS, as more difficulties are encountered when recalling meaningful stories *vs.* a word-list (Woodin et al., 2001).

Besides, VPA performance was found to depend on different strategies according to the nature of the words presented. Clark, Monk, and Maguire (2020) suggested that healthy participants mostly used verbal strategies for abstract word pairs, whereas their dominant strategy for concrete word pairs was scene visual imagery (e.g. constructing a visual image of both words within one single scene). As this strategy pertains to the visual domain, we can infer that the well-documented deficits in visuo-spatial skills in 22q11.2DS may have led to reduced use of this specific strategy, thereby affecting learning performance in our VPA task. This assumption is consistent with Maeder et al. (2020), who reported low strategy use, with mental imagery not being employed when learning single concrete words. Furthermore, constructing scene imagery constitutes a core hippocampal function (Maguire & Mullally, 2013), which aligns with concrete VPA being recognized as hippocampal-dependent (Clark et al., 2018). Therefore, it was pertinent to examine verbal learning in relation to hippocampal development in this study given that individuals with bilateral hippocampal damage show impaired scene visual imagery leading to impairments in concrete VPA, and that hippocampal atrophy has been shown in 22q11.2DS (Mancini et al., 2019).

### Volumetric differences in left hippocampal tail between 22q11.2DS subgroups

Developmental trajectories of hippocampal volume were investigated in 22q11.2DS subgroups with lower *vs.* higher verbal learning performance. While volumetric differences in several hippocampal subfields were expected given the prominent involvement of the hippocampus in memory encoding (Paller & Wagner, 2002), significant differences were revealed in the left hippocampal tail only, with a lower volume and a smaller increase with age found in the subgroup with lower performance. Though our hypothesis is only partially supported, reduced hippocampal tail volume is a consistent finding in 22q11.2DS (Mancini et al., 2019). Previous studies comparing the anterior and posterior hippocampus also corroborate our result by highlighting the role of the left posterior hippocampus in learning concise verbal information. First, the posterior hippocampus supports fine-grained memory whereas the anterior hippocampus supports coarse memory (Sekeres, Winocur, & Moscovitch, 2018). Second, Lin et al. (2017) report a power increase in slow-theta activity in the posterior hippocampus during successful encoding in a verbal memory task. Third, our result is commensurate with earlier work showing the involvement of the left hippocampus in verbal memory (Dolan & Fletcher, 1997). Finally, Sawyer et al. (2020) indicate that reduced posterior hippocampus volume is related to deficits in spatial processing. Interestingly, this finding supports our assumption above about the visuo-spatial component of VPA tasks, as the reduced left tail volume might be linked to an inadequate use of visuo-spatial strategies in participants with lower learning performance.

Another possible explanation lies in the relationship between the hippocampus and idiopathic schizophrenia, considering the increased risk of psychosis in 22q11.2DS. One cross-sectional study comparing hippocampal subfields volume in individuals with recent-onset *vs.* chronic schizophrenia found positive correlations between left hippocampal tail volume and onset age (Sasabayashi et al., 2021). Furthermore, structural and functional MRI studies found reductions in volume and in activity in the hippocampal tail in participants with schizophrenia (Maller et al., 2012; Ragland et al., 2017, respectively). Such findings thus provide insight into the implication of the hippocampal tail in the development of psychosis.

### Deterioration of verbal learning skills in participants with a psychotic spectrum disorder

We explored the development of verbal learning skills in subgroups of participants with *vs.* without PPS and with *vs.* without PSD. To our knowledge, we are the first that investigated verbal learning in relation to psychosis in 22q11.2DS using two different clinical tools. While using the traditional threshold for psychosis risk (i.e. score of ⩾3 on any SIPS positive subscale) did not lead to different verbal learning trajectories, using DSM-IV criteria to detect PSD did yield significant results. This is in line with previous findings in idiopathic schizophrenia, where individuals with the greatest cognitive decline during adolescence displayed the most severe psychotic symptoms (Dickinson et al., 2020; Mollon, David, Zammit, Lewis, & Reichenberg, 2018). Interestingly, Mancini et al. (2019), propose an association between disease progression and decreasing hippocampal volume in 22q11.2DS, which we found to be linked with poorer verbal learning performance. Thus, when comparing PSD + *vs.* PSD- participants, divergent verbal learning trajectories were revealed. We can infer that 22q11.2DS participants who will develop a psychotic disorder show significantly impaired verbal learning skills as early as 11 years. While early adolescence was found to be a critical period for the emergence of positive psychotic experiences (Chawner et al., 2019), the age of 11 also strikingly appears as a starting point of the VIQ decline in 22q11.2DS individuals with psychosis (Vorstman et al., 2015). Our study yet provides a more specific insight into the cognitive decline observed in schizophrenia. Indeed, the deterioration of verbal learning over time in PSD + participants remained significant after controlling for verbal reasoning, implying its independence from the VIQ decline observed in Vorstman et al. (2015). We thus provide evidence for verbal learning decline as a novel cognitive marker for psychosis in 22q11.2DS. Furthermore, our result is supported by Carrión et al. (2018), who found greater verbal learning deficits in individuals at clinical high-risk who transitioned to psychosis compared to individuals who did not. Such impairments significantly predicted transition to psychosis, unlike global cognitive deficits. The authors underline the importance of investigating specific cognitive impairments as trait risk markers in order to implement more targeted interventions.

### Limitations, clinical implications, and future directions

This study carries two main limitations. First, due to variations in the VPA task between scales, trajectories of verbal retention were only examined from childhood to adolescence, revealing similar age-related improvement in 22q11.2DS and HC. As this result contrasts with the lag observed for verbal learning, verbal retention skills might also develop at a slower pace but only from mid to late adolescence, which therefore could not be captured in this limited age window. Second, though the proportion of PSD + participants (16%) was almost comparable to that reported in emerging adults (Schneider et al., 2014a; 24%), our subsample size was too small to explore hippocampal development in PSD + participants alone. Future studies with larger sample sizes might address this issue.

Regarding clinical implications, our findings challenge the common view that, to acquire knowledge, 22q11.2DS individuals should rely on the verbal modality, given their visuospatial deficits (Maeder et al., 2021). More nuanced recommendations are needed to support verbal learning in 22q11.2DS. Encoding strategies should indeed be tailored to the nature of verbal stimuli. While for abstract material, verbal strategies are mostly used (e.g. verbal rehearsal), for concrete information, visual strategies are more prevalent (e.g. mental imagery; Clark et al., 2020). Additionally, assessing verbal learning skills from childhood may better identify individuals at higher risk for developing psychosis. Interestingly, individuals with schizophrenia tend to encode words using less semantic clustering (i.e. semantic categories) than serial clustering (i.e. in order of presentation), whereas the former was associated with improved verbal recall performance (Gsottschneider et al., 2011). Special attention to strategy use must thus be paid when assessing verbal memory in higher-risk patients.

## Conclusion

By investigating developmental trajectories of verbal learning skills in 22q11.2DS *vs.* HC in a broad age range, our study revealed developmental lags in deletion carriers, thereby contrasting with previous research reporting preserved verbal memory abilities. From a neuroimaging perspective, we identified that participants with lower verbal learning performance showed lower left hippocampal tail volume, with a smaller increase with age. From a psychiatric perspective, we highlighted divergent verbal learning trajectories when comparing participants with *vs.* without severe PPS. This deterioration of verbal learning skills may therefore represent a novel cognitive marker for the development of PPS in 22q11.2DS.

## Supplementary material

For supplementary material accompanying this paper visit https://doi.org/10.1017/S0033291722001842. click here to view supplementary material
