# Supplementary material for: Altered developmental trajectories of verbal learning skills in 22q11.2DS: associations with hippocampal development and psychosis
Source: Psychol Med. 2022 Jul 1;53(11):4923–32. doi: 10.1017/S0033291722001842 (PMC10476015; doi:10.1017/S0033291722001842)
Supplement: Supplementary file 1 [file S0033291722001842sup001.docx]

**Supplementary material**

**Appendix 1.**

Table A1. Participant characteristics (22q11.2DS and HC groups).

|  | | Diagnostic group | |  | Comparison | |  |  |  |
| --- | --- | --- | --- | --- | --- | --- | --- | --- | --- |
|  | 22q11.2DS | | HC |  | Pearson’s Chi square | Mann Whitney  test | | t test | *p* value |
| N at first evaluation (% of full sample) | 173 (52.1%) | | 159 (47.9%) |  |  | |  |  |  |
| Gender (N male(%)) | 86 (49.7%) | | 76 (47.8%) |  | 0.121 | |  |  | 0.728 |
| Age at first evaluation (mean(SD)) | 11.79 (4.66) | | 12.44 (5.15) |  |  | | 12943 |  | 0.354 |
| FSIQ at first evaluation (mean(SD)) | 72.01 (12.89) | | 111.5 (12.88) |  |  | |  | 27.90 | **<0.001** |
| Significant p-values (p<0.05) are shown in **bold** | | | | | | | | | |

**Appendix 2.**

For the learning phase, the participant was asked to listen and learn a list of word pairs. The first word of each pair was then given. The participant was asked to recall the second word and was provided with feedback, i.e., the correct word was given in case of incorrect or forgotten answer. This procedure was repeated during several trials. The amount of word pairs presented and the number of trials vary according to the memory scale used. Differences between scales are available in Table A2. To allow comparability between scales, a learning percentage was computed from raw scores. We divided the sum of word pairs recalled correctly across all trials with the total number of word pairs to be recalled. For the immediate recall, occurring just after the learning phase, participants were asked to freely recall as many word pairs as possible, without cuing or being given feedback. As this subscore is absent from the WMS, only the CMS subscores were included. The delayed recall then occurred approximately 30 minutes after the immediate recall. Again, only CMS subscores were taken into consideration due to different retrieval conditions between child and adult scales (with respectively free and cued recall). Both CMS subscores were available for 82% of the full sample. Similar to the learning percentage, retention percentages were computed for each recall by combining data from different test versions. The use of such percentages to account for memory performance is in line with previous work from our lab (Maeder et al., 2020, 2021b).

The flowchart below (Figure A1) illustrates the process of data analysis according to the four different memory scales.

Table A2. Total word pairs and number of trials in the learning phase of the Verbal Paired Associates subtest across memory scale versions.

| Memory scale version | | | | |  |
| --- | --- | --- | --- | --- | --- |
|  | CMS  (5-8 years) | CMS  (9-16.11 years) | | WMS-III  (from 17 years) | WMS-IV  (from 17 years) |
| Word pairs per trial | 10 | | 14 | 8 | 14 |
| Number of trials | 3 | | 3 | 4 | 4 |
| Maximum raw score | 30 | | 42 | 32 | 56 |


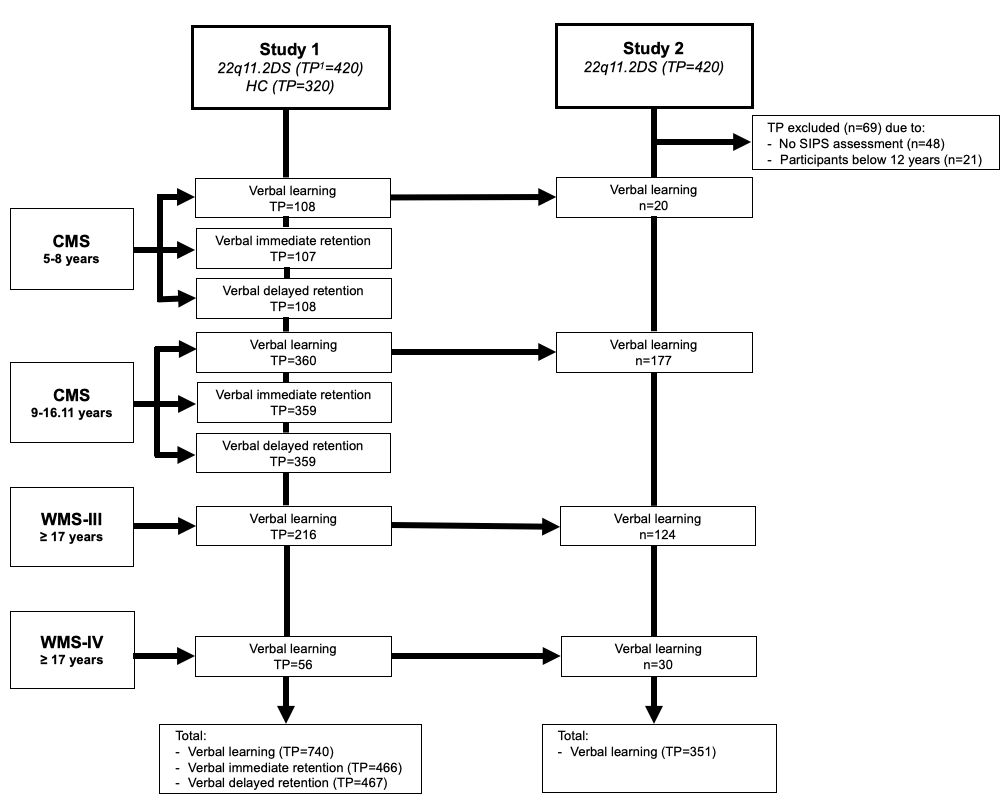


Figure A1. Flowchart of data analysis for Study 1 and Study 2, according to memory scale versions. TP, number of time-points; CMS, Children’s Memory Scale (Cohen, 1997); WMS-III and WMS-IV, Wechsler Memory Scale (Wechsler, 1997, 2009).

**Appendix 3.**

An average developmental curve of verbal learning was estimated for all participants with mixed-model regression analyses as detailed in the main text. For each participant, the individual developmental trajectory was estimated in case of more than one time-point, while if the participant had only one-time point, the value itself was taken. Then, for each participant the difference between the observed and the average group trajectory or, in the case of participants with only one time-point, the difference between the individual value and the group intercept was calculated as a summary measure indicating the deviation of each subject from the predicted developmental curve, resulting in either higher or lower verbal learning scores.

**Appendix 4**

Table A3. Results of the mixed-model regression analyses of verbal learning, verbal immediate retention, and verbal delayed retention in 22q11.2DS and HC.

|  | | | Group effect | | | | Interaction with age | | | |
| --- | --- | --- | --- | --- | --- | --- | --- | --- | --- | --- |
| Domain | Variable | Model  order | p-value | Log likelihood (df) | Intercept 22q11.2DS | Intercept  HC | p-value | Log likelihood (df) | Age slope 22q11.2DS | Age slope  HC |
| Verbal learning | Learning percentage | quadratic | **<0.001** | 86.96, 3 | 27.20 ± 6.13 | 20.71 ± 5.24 | **<0.001** | 23.92, 2 | 4.04 ± 0.83 | 3.88 ± 0.68 |
| Verbal immediate retention | Immediate retention percentage | linear | **<0.001** | 66.51, 2 | 34.58 ± 4.36 | 14.59 ± 3.99 | 0.534 | 0.39, 1 | 2.35 ± 0.37 | 2.66 ± 0.33 |
| Verbal delayed retention | Delayed retention percentage | linear | **<0.001** | 44.28, 2 | 27.68 ± 4.56 | 14.76 ± 4.15 | 0.904 | 0.01, 1 | 2.19 ± 0.38 | 2.13 ± 0.35 |
| Significant p-values (p<0.05) are shown in **bold** | | | | | | | | | |  |

**Appendix 5**

Figure A2. Comparison in developmental trajectories between 22q11.2DS and HC participants for verbal learning performance with verbal reasoning performance as a covariate.

**Appendix 6**

Table A4. Results of the mixed-model regression analyses of left tail volume in 22q11.2DS participants with higher and lower verbal learning performance.

**Appendix 7**

Table A5. Participant characteristics (PPS+ and PPS- subgroups).

|  | | | Group effect | | | | Interaction with age | | | |
| --- | --- | --- | --- | --- | --- | --- | --- | --- | --- | --- |
| Domain | Variable | Model order | p-value | Log likelihood (df) | Intercept  High | Intercept  Low | p-value | Log likelihood (df) | Age slope  High | Age slope  Low |
| Hippocampal subfield | Left tail volume | quadratic | **<0.001** | 18.01, 3 | 270.89 ± 22.62 | 283.72 ± 21.99 | **0.001** | 14.96, 2 | 11.59 ± 2.94 | 9.11 ± 2.53 |
| Significant p-values (p<0.05) are shown in **bold** | | | | | | | | | |  |

|  | | Diagnostic group | |  | Comparison |  |  |  |
| --- | --- | --- | --- | --- | --- | --- | --- | --- |
|  | PPS+ | | PPS- |  | Pearson’s Chi square | Mann Whitney  test | t test | *p* value |
| N at first evaluation (% of full sample) | 74 (62.2%) | | 45 (37.8%) |  |  |  |  |  |
| Gender (N male(%)) | 36 (48%) | | 25 (55.6%) |  | 0.534 |  |  | 0.465 |
| Age at first evaluation (mean(SD)) | 12.45 (4.26) | | 13.30 (4.22) |  |  | 1385 |  | 0.126 |
| FSIQ at first evaluation (mean(SD)) | 71.36 (11.94) | | 72.06 (12.12) |  |  |  | 0.31 | 0.756 |
| Significant p-values (p<0.05) are shown in **bold** | | | | | | | | |

**Appendix 8**

Table A6. Participant characteristics (PSD+ and PSD- subgroups).

|  | 22q11.2DS group | | | Comparison | |  | |  |  | |
| --- | --- | --- | --- | --- | --- | --- | --- | --- | --- | --- |
|  | PSD+ | PSD- | Pearson’s  Chi square | | Mann Whitney  test | | t test | | *p* value |  |
| N at first evaluation (% of full sample) | 19 (16%) | 100 (84%) |  | |  | |  | |  |  |
| Gender (N male(%)) | 13 (68.4%) | 48 (48%) | 6.429 | |  | |  | | 0.169 |  |
| Age at first evaluation (mean(SD)) | 14.15 (4.76) | 12.52 (4.12) |  | | 740 | |  | | 0.129 |  |
| FSIQ at first evaluation (mean(SD)) | 66.37 (10.69) | 72.6 (11.98) |  | |  | | 2.123 | | **0.037** |  |
| Significant p-values (p<0.05) are shown in **bold** | | | |  | |  | |  |  | |

**Appendix 9**

Table A7. Results of the mixed-model regression analyses of verbal learning in PPS+ and PPS- subgroups.

|  | | | Group effect | | | | Interaction with age | | | |
| --- | --- | --- | --- | --- | --- | --- | --- | --- | --- | --- |
| Domain | Variable | Model  order | p-value | Log likelihood (df) | Intercept PPS+ | Intercept  PPS- | p-value | Log likelihood (df) | Age slope  PPS+ | Age slope  PPS- |
| Verbal learning | Learning percentage | quadratic | 0.150 | 4.82, 3 | 44.27 ± 12.21 | 17.70 ± 7.79 | 0.113 | 4.04, 2 | 1.08 ± 1.48 | 4.31 ± 0.95 |
| Significant p-values (p<0.05) are shown in **bold** | | | | | | | | | |  |

**Appendix 10**

Table A8. Results of the mixed-model regression analyses of verbal learning in PSD+ and PSD- subgroups.

|  | | | Group effect | | | | Interaction with age | | | |
| --- | --- | --- | --- | --- | --- | --- | --- | --- | --- | --- |
| Domain | Variable | Model  order | p-value | Log likelihood (df) | Intercept PSD+ | Intercept  PSD- | p-value | Log likelihood (df) | Age slope  PSD+ | Age slope  PSD- |
| Verbal learning | Learning percentage | quadratic | **<0.001** | 25.99, 2 | 44.05 ± 3.02 | 66.30 ± 6.73 | **<0.001** | 22.16, 1 | 0.84 ± 0.19 | -1.11 ± 0.40 |
| Significant p-values (p<0.05) are shown in **bold** | | | | | | | | | |  |

**Appendix 11**

To examine longitudinal correlations between verbal learning performance and PPS severity, we used the *fitlme* function in MATLAB. We tested the associations between verbal learning performance and the severity scales of the five PPS listed previously. The results were covaried for age and adjusted for multiple comparison using FDR correction. We first found a significant negative correlation between verbal learning performance and the sum of the severity scales of the five PPS (p=0.014, R=−0.168). Another significant correlation was found between verbal learning performance and the P1 subscale (p=0.014, R=−0.173). Therefore, these findings support and extend our results from the mixed-model regression analysis presented in Appendix 8, Table A10. While they first indicate that lower verbal learning performance is correlated with more severe PPS, they further suggest that poorer skills are especially associated with experiencing unusual thought content and delusional ideation (P1).
